# Effects of Large-Scale Releases on the Genetic Structure of Red Sea Bream (*Pagrus major*, Temminck et Schlegel) Populations in Japan

**DOI:** 10.1371/journal.pone.0125743

**Published:** 2015-05-20

**Authors:** Enrique Blanco Gonzalez, Masato Aritaki, Halvor Knutsen, Nobuhiko Taniguchi

**Affiliations:** 1 The Research Institute of Marine Bioresources, Fukuyama University, Onomichi, 722-2101, Japan; 2 University of Agder, N-4604, Kristiansand, Norway; 3 Institute of Marine Research (IMR), Flødevigen, N-4817, His, Norway; 4 Coastal Fisheries and Aquaculture Division, Seikai National Fisheries Research Institute, Fisheries Research Agency, Nagasaki, 851-2213, Japan; 5 Centre for Ecological and Evolutionary Synthesis (CEES), Department of Biosciences, University of Oslo, Blindern, N-0316, Oslo, Norway; College of Charleston, UNITED STATES

## Abstract

Large-scale hatchery releases are carried out for many marine fish species worldwide; nevertheless, the long-term effects of this practice on the genetic structure of natural populations remains unclear. The lack of knowledge is especially evident when independent stock enhancement programs are conducted simultaneously on the same species at different geographical locations, as occurs with red sea bream (*Pagrus major*, Temminck et Schlegel) in Japan. In this study, we examined the putative effects of intensive offspring releases on the genetic structure of red sea bream populations along the Japanese archipelago by genotyping 848 fish at fifteen microsatellite loci. Our results suggests weak but consistent patterns of genetic divergence (*F*_ST_ = 0.002, *p* < 0.001). Red sea bream in Japan appeared spatially structured with several patches of distinct allelic composition, which corresponded to areas receiving an important influx of fish of hatchery origin, either released intentionally or from unintentional escapees from aquaculture operations. In addition to impacts upon local populations inhabiting semi-enclosed embayments, large-scale releases (either intentionally or from unintentional escapes) appeared also to have perturbed genetic structure in open areas. Hence, results of the present study suggest that independent large-scale marine stock enhancement programs conducted simultaneously on one species at different geographical locations may compromise native genetic structure and lead to patchy patterns in population genetic structure.

## Introduction

Spatial patterns in genetic structure of organisms in the wild reflect historical and contemporary effects of micro-evolutionary processes (e.g., genetic drift, gene flow, mutation, selection) acting on animal and plant populations. Intrinsic life-history features and differences in environmental regimes are also relevant to genetic population structuring. The evolutionary and adaptive potential of the species to cope with new environmental conditions demands such intra-specific diversity (e.g., [1]). While gene flow promotes genetic homogeneity, genetic drift and especially directional selection tend to segregate populations [2–4]. In fishes, genetic divergence among populations of marine species is often less pronounced than for freshwater and anadromous species [5, 6]. Those differences have been ascribed to larger population sizes and higher connectivity and gene flow potential in marine species, which could mask random mutation and drift effects. In contrast, divergence in relation to selective forces can be more significant and act at a faster pace [7, 8]. Three different types of adaptive responses have been proposed: 1) short-term adaptive responses via phenotypic plasticity; 2) long-term adaptation by shift in habitat use; and 3) adaptation via genic selection [4, 7]. Thus, major challenges in conservation and evolutionary biology reside in understanding the interplay between the different mechanisms shaping the genetic structuring within a species, and recognizing the future implications of adaptive population divergence (e.g., [1]).

In addition to natural processes, the genetic structure and adaptive potential of marine populations can be subject to significant human-induced stressors. One of the most controversial activities is associated with the putative harmful genetic effects of translocations and hatchery-produced juvenile releases on native populations [3, 9–13]. Although such releases have proven very useful for sustaining fishing yields [14–16], they also may represent a serious threat to gene-level biodiversity even over just a few generations [8, 12, 13]. Hatchery-released juveniles usually are produced from a small broodstock who harbor only a small portion of the genetic diversity within a species. Interbreeding between released and wild specimens may alter the genetic composition of indigenous populations. Additionally, selective forces acting on offspring reared in captivity differ significantly from those faced by wild populations, which may lower subsequent reproductive success [8, 17]. Differences in genetic composition and fitness would magnify in case interactions occur with hatchery individuals originated from selective breeding programs, i.e., escapees from commercial farms. It is noteworthy, though, that most studies on population fitness have been conducted upon salmonids, with little emphasis on other marine species [11–13] and references therein. Major efforts have been made to maximize the gene pool of hatchery offspring and minimize their differences with native populations [3, 17, 18] and references therein; however, implementation of those practices on large-scale programs is still uncommon.

Both high survival and high recapture rates are needed to justify the economic investment in marine stock enhancement programs. Thus, they usually are conducted in semi-enclosed embayments and on species not displaying highly migratory behavior [14, 15]. These strategies can contribute to increase recaptures; nevertheless, they also can render small local native populations more vulnerable than in the open sea, where dispersal and gene flow possibilities are higher. In this regard, while most studies have addressed the genetic effects of juvenile releases for specific programs at specific locations, little is known about the genetic integrity of large marine populations when several independent stock enhancement programs are conducted on the same species at different locations across its distribution range [11]. In Japan, for example, many commercially important marine species are intensively released as part of stock enhancement programs carried out by prefectural governments. Sometimes, several prefectures run independent programs on the same species simultaneously [14, 19]. The performance and genetic assessment of each program usually is uncoordinated and evaluated independently, overlooking issues of connectivity and genetic structure at a larger scale.

**Fig 1:**
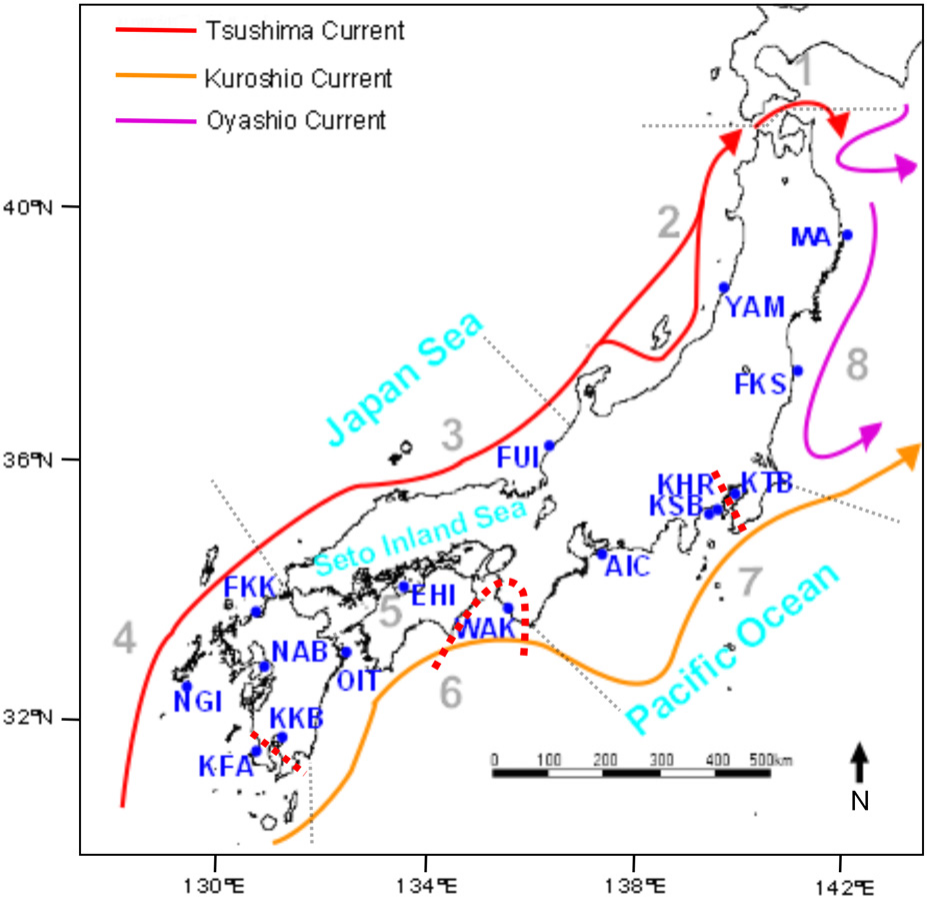
Sampling locations (solid circles, for details see Table 1) and main circulation features associated with major water masses around the Japanese archipelago. Broken red lines indicate the location of the three major barriers to gene flow inferred based on the microsatellite data. Broken grey lines delimit the eight coastal regions dividing the country: 1. Hokkaido, 2. Northern Japan Sea, 3. Western Japan Sea, 4. East China Sea, 5. Seto Inland Sea, 6. Southern Pacific, 7. Central Pacific, 8. Northern Pacific.

Ocean dynamics along the Japanese archipelago is dominated by three main strong currents (see Fig 1), which offer high potential for pelagic dispersal by oceanic drift [20] and references therein, [21]. On the eastern coast, the warm Kuroshio Current flows northeastward along the Pacific coast. Meanwhile, the warm Tsushima Current, a branch of the Kuroshio Current, flows northeastwards through the Tsushima Strait and along the Japan Sea coast and the western coast of the main island of Honshu. A coldwater front, the Oyashio Current, flows southwards along the northern island of Hokkaido and the northeastern-most part of the main island of Honshu.

Red sea bream (*Pagrus major*) was one of the eight species selected during the early development of marine stock enhancement in Japan [14]. Since then, millions of juveniles of this species have been released along the coasts of most coastal prefectures in the country, representing in number, the second-highest release of a marine fish species in Japan [22]. However, genetic studies of the effects of stock enhancement mostly have been conducted in two prefectures, Kagoshima and Kanagawa. In both cases, significant differences among the gene pools of released offspring and native wild populations were reported [23–25]. Red sea bream in Japan is believed to comprise a single panmictic population [19, 26–28]. However, only the early study on allozyme markers conducted by Taniguchi and Sugama [19] included samples from Kagoshima and Kanagawa prefectures. The remaining studies screened only a few samples from the central and southern part of the country, using few markers with limited resolution power, e.g. D-loop mtDNA sequencing [26] and three microsatellite loci [27, 28].

The present study represents the most comprehensive analysis of population genetic structure of red sea bream in Japan by genotyping 848 specimens from fifteen sampling localities and one recapture sample (Fig 1) at fifteen microsatellite loci. In addition to characterizing the genetic resources of the species in the country, we investigated whether independent large-scale stock enhancement programs conducted simultaneously on the same species at different geographical locations may perturb the genetic structure of red sea bream in Japan; and if so, whether the effects are restricted to semi-enclosed areas or whether could they also be detected in more open areas.

### The species and its release history in Japan

Red sea bream belongs to the family Sparidae. This demersal fish is widely distributed in coastal waters of the Northwest Pacific extending northwards to Hokkaido in Japan, excluding the Ryukyu Islands, and southwards to the southern part of the Korean Peninsula, the East China Sea, the South China Sea, and Taiwan [29]. It usually occurs over rocky substrates, but also inhabits areas with soft sandy and muddy bottoms and reefs at 10 to 200 m depths. It can attain 100 cm in standard length (SL) and live more than 10 years. Red sea bream is a protogynous hermaphrodite, i.e., individuals are born as females, and later switch to male; most of the fish attain maturity 4 years after birth, beyond 30 cm in SL [30, 31]. In spring, depending on sea water temperature, adults migrate into shallower areas to spawn [32]. In Japan, the main spawning grounds have been proposed to be on the southern island of Kyushu, with smaller spawning areas in the Japan Sea, the Seto Inland Sea and the Pacific Ocean [19] and references therein, [33]. During the spawning season, a mature female may release a few million eggs divided into several batches over a month-long period [31, 34]. Eggs and larvae are transported with ocean currents, and larvae progressively shift their mode of life from pelagic to demersal [35]. Juvenile red sea bream use sandy bottom covered with seagrass as nursery grounds and refuge against predators [36]. In winter, red sea bream swim offshore to deeper waters for over-wintering [37].

**Fig 2:**
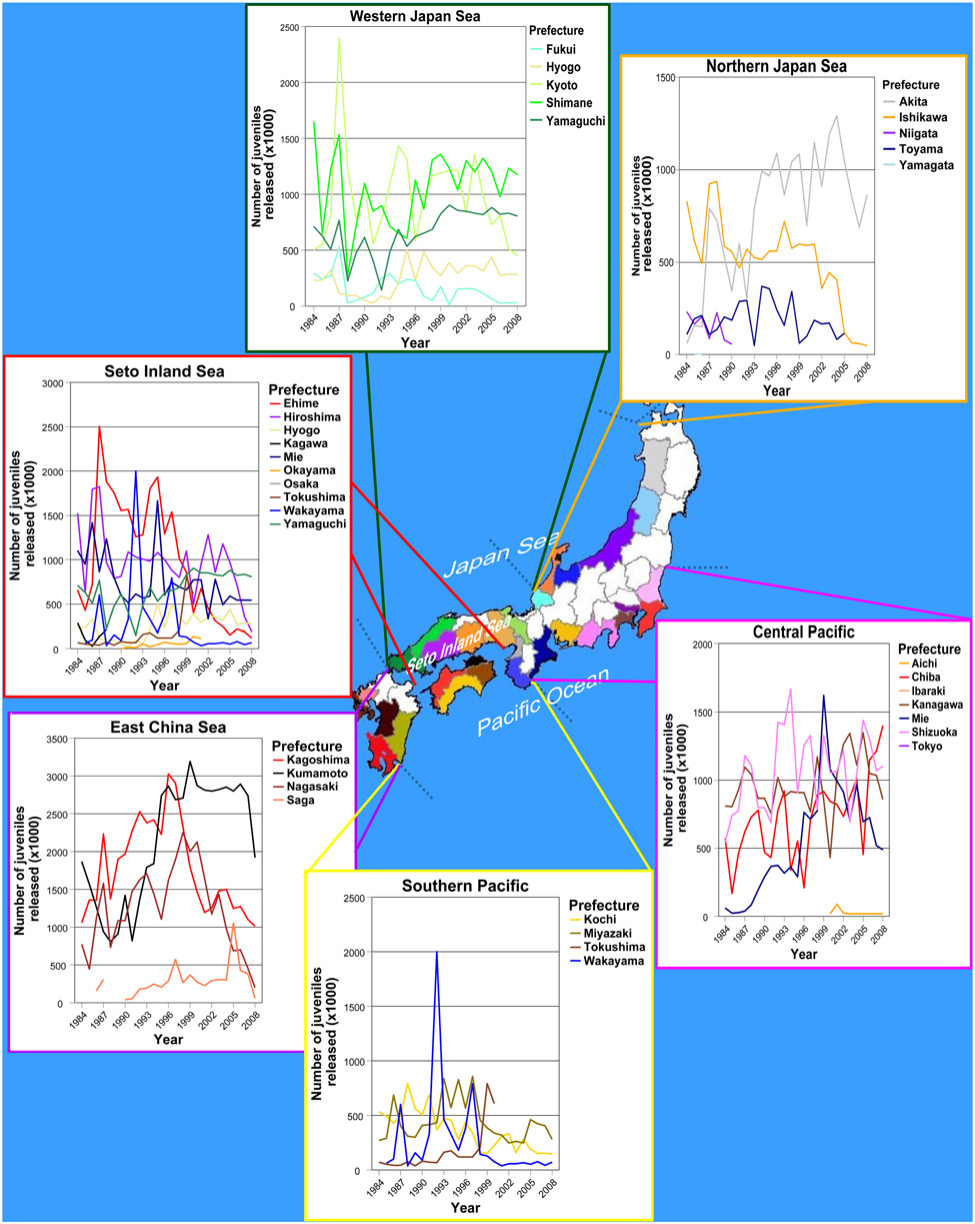
Annual variation in number of red sea bream juveniles releases (x1000) by prefectures (colored areas) and regions (dashed grey lines). For each region, colors of the prefectures in the map correspond to those used to represent the annual variation in number of releases. Note that no releases have been performed in Hokkaido and the Northern Pacific region.

In Japan, red sea bream, commonly called “madai” “tai", is one of the most important commercial and sport fishing species. The high demand and price of this species is related to the Japanese culture [38]; in fact, its characteristic whitish/reddish coloration has been traditionally associated with good fortune, beauty, festivals and celebrations. In 1963, it was chosen as one of the target coastal species to be released in the Seto Inland Sea of Japan [14]. Since then, more than 500 million hatchery-reared juveniles have been released in Japan, throughout the majority of coastal prefectures ([22], Fig 2), representing the second largest species in terms of number of juveniles released in Japan after Japanese flounder (*Paralichthys olivaceus*). Stock enhancement programs for red sea bream in Japan are run regionally, usually at hatcheries operated by prefectural governments. The size, origin and replacement rate of the broodstock as well as the number of juveniles released every year are specific for each program. The largest numbers of annual juvenile releases were reported in the East China Sea, in the southern prefectures of the island of Kyushu, e.g., Kagoshima and Kumamoto (Fig 2). The Seto Inland Sea and the Sagami Bay-Tokyo Bay complex around Kanagawa Prefecture near Tokyo, on the Pacific coast, are other areas receiving large numbers of released juveniles annually. In contrast, most northern prefectures decided not to conduct juvenile red sea bream releases. The northwestern prefecture of Akita appears as an exception, accounting for 500,000–1 million juvenile releases annually.

## Materials and Methods

### Fish sampling and microsatellite genotyping

In 2007 and 2008, a total of 848 adult red sea bream were collected at fifteen sampling localities along the Japanese archipelago (details provided in Table 1 and Fig 1): Aichi (AIC), Fukui (FUK), Yamagata (YAM), Fukushima (FKS), Iwate (IWA), Kanagawa Sagami Bay (KSB), Kanagawa Tokyo Bay (KTB); Wakayama (WAK); Ehime (EHI); Fukuoka (FKK), Nagasaki Ariake Bay (NAB), Nagasaki Goto Island (NGI), Oita (OIT), Kagoshima Kinko Bay (KKB) and Kagoshima Fukiagehama (KFA). In addition, some fish from Kanagawa Sagami Bay presented the characteristic deformity of the inter-nostril epidermis developed in hatchery fish [39]. Thereafter, these individuals were considered as recaptured hatchery-released specimens (KANHR). A small part of the data set was used previously to develop the microsatellite multiplex PCR panels [40] and to characterize the genetic resources in Kanagawa Prefecture [25], although here we provide full data set to perform the complete analysis along the Japanese archipelago.

**Table 1:** Sample location, abbreviation (ID), latitude, longitude, sample size (*n*), number of alleles (*A*), allelic richness (*A*_r_, n = 26), heterozygosity (*H*_S_), *F*_IS_ and *p*-value.

Location	ID	Latitude	Longitude	*n*	*A*	*A*_r_	*H*_S_	*F*_IS_	*p*-value	HW deviating loci
Aichi	AIC	34.4	137.0	60	18.8±9.5	14.6±6.8	0.811	**0.057**	0	*Pma*103-59*NCL*
Fukui	FUK	36.1	136.0	60	20.2±8.7	15.2±6.6	0.817	**0.041**	0	
Yamagata	YAM	38.3	139.3	60	19.6±9.5	15.1±6.8	0.827	**0.015**	0.003	
Fukushima	FKS	37.4	141.1	40	17.9±7.6	15.3±6.3	0.830	**0.055**	0	*Pma*4-32*NCL*
Iwate	IWA	39.3	142.1	60	19.0±8.1	14.7±6.1	0.823	**0.054**	0	*Pma*22-9*NCL*
Kanagawa Sagami Bay	KSB	35.1	139.2	38	17.7±7.8	15.3±6.7	0.831	**0.038**	0.010	*Kpm*25, *Pma*22-9*NCL*
Kanagawa Tokyo Bay	KTB	35.2	139.4	40	15.9±7.0	14.2±6.1	0.821	**0.045**	0	
Kanagawa Hatchery-Released	KHR	35.1	139.2	26	12.8±5.6	12.8±5.6	0.809	**0.053**	0.003	
Wakayama	WAK	33.4	135.1	44	16.7±7.8	14.0±6.4	0.813	**0.063**	0	*Pma*5
Ehime	EHI	34.1	133.1	60	20.9±8.7	15.7±6.4	0.831	**0.029**	0	
Fukuoka	FKK	32.3	130.2	60	20.1±9.9	15.3±7.2	0.821	**0.032**	0.002	
Nagasaki Ariake Bay	NAB	33.4	130.1	60	19.6±8.6	15.0±6.3	0.823	**0.037**	0.001	
Nagasaki Goto Island	NGI	32.3	128.5	60	20.3±9.8	15.5±7.1	0.827	**0.010**	0.007	
Oita	OIT	33.1	131.6	60	19.3±8.3	14.9±6.1	0.815	**0.039**	0	*Pma*22-9*NCL*, *Pma*4-32*NCL*
Kagoshima Kinko Bay	KKB	31.4	130.4	60	19.1±7.9	14.3±6.1	0.803	0.019	0.100	*Pma*22-9*NCL*, *Pma*18-41*N*CL
Kagoshima Fukuagehama	KFA	31.3	130.1	60	20.3±8.5	15.3±6.6	0.822	**0.039**	0	

Loci deviating from Hardy-Weinberg proportions (either direction) are given in parenthesis (without adjustment for multiple tests). Bold values of *F*_IS_ denote significant values in either direction.

Red sea bream samples were collected by gillnets, immediately transferred to a seawater tank, and sacrificed in the most gentle and swift way by percussive stunning with a priest. Practices for present sampling and handling of fish for this study were approved by the Animal Ethics Committee of Fukuyama University and were performed by experienced personnel. The species is not protected by Japanese law when sampling was performed (it is a commercially harvested species in Japan), and no special permits were required for sampling the species either for research or commercially at these locations (see details in Table 1). Whole sacrificed fish were immediately frozen and stored at -20°C until reaching laboratory facilities, where fin tissue was taken and placed into 96% ethanol prior to DNA extraction.

Total genomic DNA was extracted from fin tissues using either the DNeasy kit (Qiagen) or the DNAzol (Invitrogen) protocol, re-suspending the DNA in TE buffer. Microsatellite polymorphism was analyzed at fifteen polymorphic markers: *Kpm*1, *Kpm*11, *Kpm*7, *Kpm*22, *Kpm*2, *Kpm*23, *Kpm*25, *Pma22-*9*NCL*, *Kpm*28, *Pma11-45NCL*, *Pma4-32NCL*, *Pma103-59NCL*, *Pma18-41NCL*, *Pma*1, *Pma*5; following the same fluorescent dye labeling and multiplex PCR protocols previously described by Blanco Gonzalez et al. [40]. Amplified PCR products were run with GeneScan^Tm^–600 Liz as size standard on an ABI 3500XL Genetic Analyzer (Applied Biosystems), and individual genotyping was analyzed with GENEMAPPER v. 4.1 (Applied Biosystems).

### Genetic diversity and differentiation

Levels of genetic variation were characterized by counting observed allele (*A*), allelic richness (*A*_r_) and gene diversity within samples (*H*_S_) and the average for all samples (*H*_T_), based on Nei and Chesser [41], using FSTAT v. 2.9.3.2 software package [42]. Deviations from Hardy–Weinberg (HW) equilibrium, including significantly higher or lower *F*_IS_ [43] estimates than expected by chance, were investigated by Fisher’s exact probability test in GENEPOP v. 4.0 [44]. We adopted the false discovery rate (FDR) approach [45] when interpreting the significance of test results. Linkage disequilibrium (LD) among all pairs of loci in all samples was tested by a Fisher’s exact test with 10,000 demorizations, 100 batches, and 1000 iterations per batch in GENEPOP v. 4.0 [44]. The presence of null alleles or technical artifacts was investigated with MICROCHECKER v. 2.2.1 [46]. Statistical evidence for selection was tested for by the outlier tests implemented in BAYESCAN [47].

Genetic differentiation among samples were quantified by Wright’s *F*_ST_, using Weir and Cockerham’s [43] estimator *θ* within all samples and also within pairs of sample localities. The statistical significance of the analysis was estimated by exact tests in GENEPOP v. 4.0 [44], with 10,000 dememorizations and batches, using 10,000 iterations per batch. The *p* values were calculated for each locus separately and summed over loci by Fisher’s summation procedure following Ryman and Jorde [48]. In this case, we adopted the Benjamini and Yukutieli's [49] FDR approach, which is applicable also to non-independent tests, when interpreting the significance of *p* values.

### Spatial structure and population admixture

Spatial genetic differentiation patterns were examined by a Principal Component Analysis (PCA) based upon a covariance matrix of allele frequencies, and visualized using PCAGEN v. 1.2.1 [50]. The Bayesian clustering method implemented in the software STRUCTURE v. 2.3.3. [51] was performed to characterize spatial patterns of genetic clusters (*K*) and to infer admixture proportions between wild and hatchery-reared fish in the data set without *a priori* information of population partition. We assumed an admixture model and correlated allele frequencies [52]. Each run consisted of a burn-in of 50,000 MCMC steps, followed by 200,000 steps, for values of *K* between 1 and 7, and the calculation was done five times for each *K*. The most likely number of clusters, *K*, was estimated as the value which maximized the averaged log-likelihood, log Pr(*X*|*K*) and the ad hoc statistic Δ*K* [53]. Once *K* was determined, individuals were assigned to the respective clusters and plotted with DISTRUCT v. 1.1 [54].

Geographic patterns and the scale at which genetic structuring occurs were further investigated by testing putative correlations between genetic and geographic distances [55]. Pairwise *F*_ST_ estimates were linearized, as *F*_ST_ ⁄ (1—*F*_ST_), and regressed against the natural logarithm of the shortest maritime distance connecting each sample pair (Fig 1). Isolation-by-distance effects were tested by a Mantel test performed in IBDWS v. 3.23 ([56], http://ibdws.sdsu.edu/~ibdws/). As sample KHR comprised only hatchery-released specimens, it was excluded in this analysis and in the subsequent landscape genetic analysis performed in BARRIER v. 2.2 [57]; this software was used to identify barriers to gene flow among locations. As input for the program, we used sample geographical coordinates and pairwise *F*_ST_ estimates along all pairs of localities. The analysis was performed including all loci to infer the rank of importance of the barriers. Statistical support for each barrier was evaluated by the number of loci that supported it, and by 1000-bootstrap analysis of the multilocus Weir and Cockerham’s [43] *F*_ST_ matrix generated using the DIVERSITY R package [58].

The amount of genetic variation explained by alternative sample grouping was tested with AMOVA using ARLEQUIN v. 3.5 [59]. As all samples included adults of different length ranges; we did not consider temporal effects in relation to the year they were collected, i.e., 2007 or 2008. We specifically tested the null hypotheses of panmixia, as well as of structuring by geographical regions (Japan Sea, Pacific Ocean and Seto Inland Sea) and by the PCA analysis results (KHR, KKB, KSB, WAK and the rest of the samples).

We estimated the effective population sizes (*N*_e_) under a sample-size bias correction [60] using the linkage disequilibrium method implemented in LDNE v. 1.31 [61]. Harmonic mean *N*_e_ and jackknife-adjusted 95% confidence intervals for each sample were estimated using Pcrit = 0.02, i.e., excluding those alleles with frequencies lower than 0.02. Waples and Do [62] recognized the limitations of the method in obtaining precise estimates when populations are large; still, they found the approach useful to discriminate between small and large populations.

## Results

### Genetic diversity and differentiation

The results are based on a total of 848 Japanese red sea bream individuals genotyped at fifteen microsatellite loci (Table 1). All loci showed high levels of polymorphism (Table 2). The lowest polymorphism was observed at locus *Kpm*1, exhibiting 6 alleles and *H*_T_ = 0.441; in contrast to locus *Pma*22-9*NCL* which exhibited 52 alleles and *H*_T_ = 0.959. Levels of genetic variability, expressed as mean allelic richness (*A*_r,_ based on minimum sample size n = 26) and gene diversity within samples (*H*_S_), appeared very similar among sample localities (Table 1); except for the hatchery-released sample from Kanagawa, KHR, which exhibited a lower number of alleles.

**Table 2:** Genetic diversity among red sea bream samples at 15 loci.

	*A*	*H*_T_	*F*_ST_	*p*-value
*Kpm*1	6	0.441	-0.002	0.588
*Kpm*11	14	0.521	0.003	**0.004**
*Kpm*7	31	0.819	0.006	**0.003**
*Kpm*22	40	0.950	0.002	0.055
*Kpm*2	44	0.958	0.002	**< 0.001**
*Kpm*23	20	0.830	0.001	**0.002**
*Kpm*25	50	0.837	0.003	**0.014**
*Pma*22-9*NCL*	52	0.959	0.002	**0.004**
*Kpm*28	41	0.950	0.001	0.248
*Pma*11-45*NCL*	28	0.919	0	0.328
*Pma*4-32*NCL*	38	0.946	0.003	**< 0.001**
*Pma*103-59*NCL*	29	0.784	0.005	**< 0.001**
*Pma*18-41*NCL*	29	0.851	0.002	**< 0.001**
*Pma*1	36	0.671	0.001	**0.013**
*Pma*5	32	0.907	0.002	**0.039**
Overall		0.823	0.002	**< 0.001**

Allele counts (*A*), total gene diversity (*H*_T_), level of genetic differentiation among samples (*F*_ST_) and exact test *p*-values for allele frequency homogeneity (Fisher’s procedure, bold font indicates significant *p*-values).

In general, loci conformed to Hardy-Weinberg (HW) expectations (Table 2). Deviation from HW equilibrium was observed in 33 (13.7%) of 240 possible cases. After FDR correction, ten of them (4.1%) remained statistically significant at the 5% level (Table 2), and all cases were attributed to deficiency of heterozygotes. Four of those ten significant cases occurred at locus *Pma*22-9*NCL*, where MICROCHECKER suggested the existence of null alleles. An examination of *F*_IS_ estimates for each allele separately in each sample did not indicate any pattern in the departure from HW genotype proportions. Omitting locus *Pma*22-9*NCL*, overall *F*_ST_ yielded essentially the same result (i.e. *F*_ST_ changed from 0.0021 to 0.0022), as expected for a locus with potential null alleles when *F*_ST_ estimates are small [63, 64]; therefore, we decided to keep data from this locus in the analyses. The remaining six cases appeared randomly distributed among loci and sample localities.

Linkage disequilibrium was found to be statistically significant (at the 5% level) in 81 out of 1680 pairwise tests (4.8%). Twenty of them (1.2%) remained statistically significant after the FDR correction, distributed evenly among samples and pairs of loci. Thus, our results provided no reason to conclude that the loci were linked. The outlier test on BAYESCAN suggested no evidence for selection operating on any of the loci (results not shown).

### Spatial structure and population admixture

Overall genetic divergence among sample localities was low, but statistically significant (*F*_ST_ = 0.002, *p* < 0.001). Differences were significant (at the 5% level) at 11 of the 15 scored loci (Table 2). Pairwise *F*_ST_ comparisons indicated significant differentiation in 64 out of 120 pairs (53%), with 45 (37%) remaining statistically significant after FDR (Table 3). Most of the statistically significant comparisons (38 out of 45) involved three samples: KHR (15 cases) and WAK and KKB (13 cases each). The remaining seven cases appeared evenly distributed among sample pairs.

**Table 3:** Estimated pairwise *F*_ST_ values (averaged over loci: below diagonal) and corresponding *p* values for tests of allele frequency differences (calculated over loci: above diagonal) among red sea bream samples at 15 microsatellite loci.

	AIC	FUK	YAM	FKS	IWA	KSB	KTB	KHR	WAK	EHI	NAB	FKK	NGI	OIT	KKB	KFA
AIC		0.441	0.914	**0.008**	0.251	**0.004**	0.177	**0.000**	**0.001**	0.019	0.583	0.547	0.608	0.017	0.061	0.694
FUK	0.000		0.401	0.286	0.060	0.149	0.054	**0.000**	**0.000**	0.782	0.406	0.544	0.559	0.016	**0.000**	0.229
YAM	-0.002	0.000		0.062	0.013	0.364	0.110	**0.000**	**0.000**	0.242	0.099	0.358	0.870	0.013	**0.001**	0.533
FKS	0.002	0.000	0.001		0.036	**0.001**	0.066	**0.000**	**0.000**	0.617	0.119	0.115	0.048	0.025	**0.000**	0.096
IWA	-0.001	0.001	0.000	0.000		0.087	0.042	**0.000**	**0.000**	**0.000**	0.072	0.010	0.118	**0.000**	**0.000**	0.041
KSB	0.002	0.002	0.000	0.003	-0.002		0.090	**0.000**	**0.000**	0.023	0.181	0.181	0.165	0.010	**0.000**	0.470
KTB	0.000	0.003	0.000	0.000	0.001	0.002		**0.000**	**0.000**	**0.008**	0.058	0.114	0.077	0.104	**0.000**	0.366
KHR	0.012	0.014	0.013	0.015	0.014	0.012	0.018		**0.000**	**0.000**	**0.000**	**0.000**	**0.000**	**0.000**	**0.000**	**0.000**
WAK	0.003	0.006	0.004	0.004	0.004	0.008	0.006	0.017		**0.000**	**0.000**	0.030	0.040	**0.000**	**0.000**	**0.001**
EHI	0.001	-0.001	-0.001	0.000	0.001	0.002	0.003	0.015	0.005		0.022	0.062	0.082	**0.005**	**0.009**	0.110
NAB	-0.001	0.001	0.000	0.001	-0.001	0.000	0.001	0.012	0.004	0.000		0.300	0.532	0.069	**0.004**	0.729
FKK	-0.001	0.001	0.001	0.001	0.000	0.001	0.002	0.013	0.001	0.000	-0.001		0.452	0.048	**0.008**	0.909
NGI	-0.001	0.001	-0.002	0.001	-0.001	0.000	0.002	0.011	0.002	0.000	-0.002	-0.001		0.045	**0.008**	0.308
OIT	0.000	0.002	0.001	0.003	0.001	0.001	0.001	0.016	0.003	0.001	-0.001	0.000	0.000		**0.000**	0.244
KKB	0.002	0.003	0.005	0.006	0.007	0.010	0.007	0.017	0.007	0.004	0.004	0.006	0.005	0.003		0.029
KFA	-0.001	0.001	-0.001	0.001	0.000	0.001	0.002	0.012	0.004	0.000	-0.002	-0.001	0.000	0.000	0.004	

Bold *p*-values are statistically significant at the 5% level after false discovery rate [49] correction (*q* = 0.0093).

**Fig 3:**
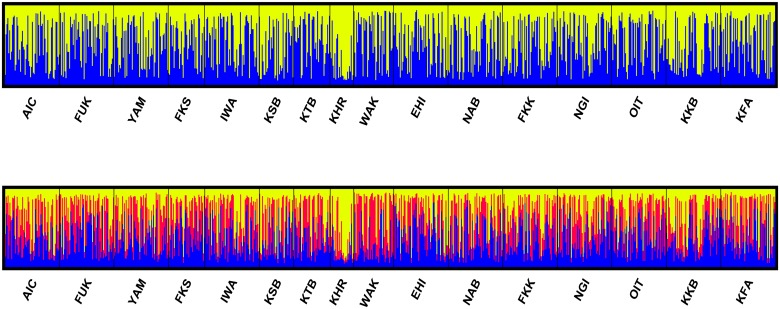
Results of the Bayesian clustering analysis of 16 red sea bream samples. Each bar denotes an individual, while colors denote clusters. Here, we show results of *K* = 2 (Pr(*X*|*K*) = -61976) and *K* = 3 (Pr(*X*|*K*) = -61854), the two most likely outcomes.

Results of the Bayesian clustering analysis performed with STRUCTURE revealed significant population genetic structure and suggested the existence of three clusters, *K* = 3. Fig 3 illustrates the admixture proportions of individuals considering *K* = 2 and *K* = 3. Considering *K* = 2, cluster 1 corresponded to the hatchery-released sample from Kanagawa, KHR, and the sample from the inner bay in Kagoshima, KKB, where the proportion of hatchery-released fish were reported to be very high [11]; while cluster 2 comprised the rest of the samples. When considering *K* = 3, WAK appeared as a third cluster, while the other samples appeared less admixed. Analyses assuming *K* > 3 did not resolve further groupings of individuals, reflecting the low level of differentiation among samples or the limitations of STRUCTURE to determine the correct number of genetic clusters when genetic differentiation among samples is low [51, 52, 65].

**Fig 4:**
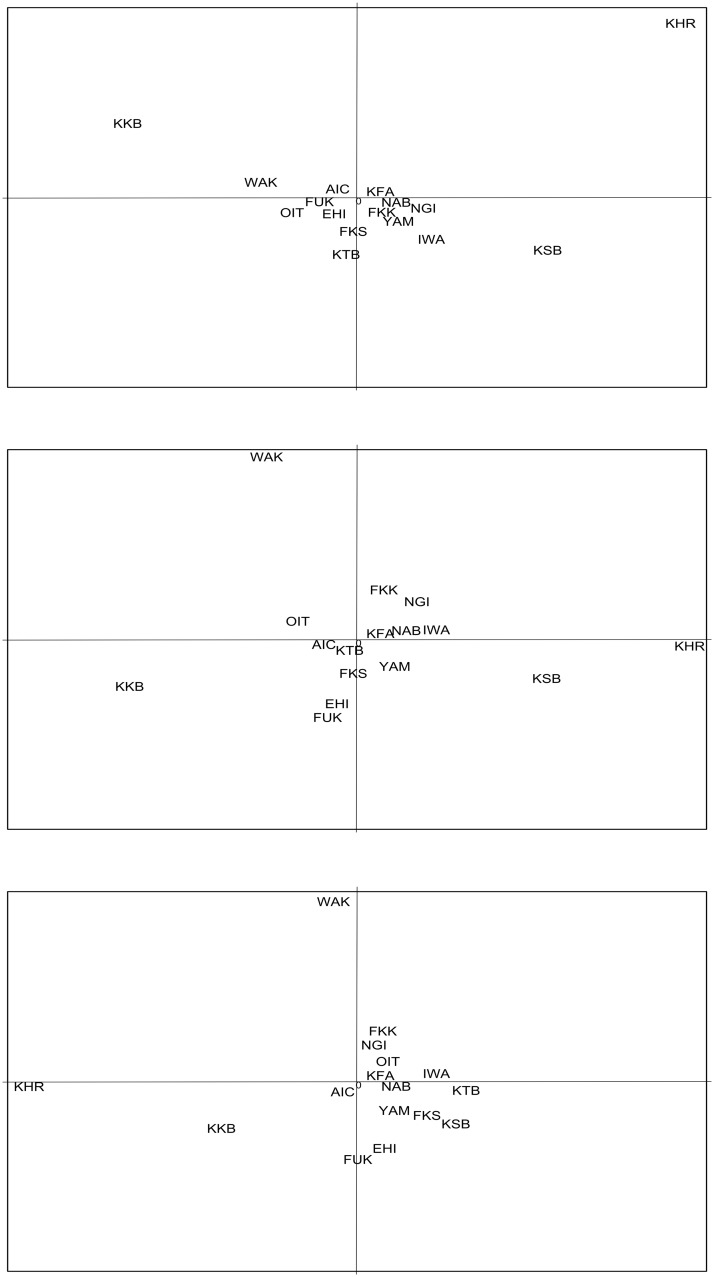
Principal Components Analysis (PCA) of allele frequencies. The plots display the three principal components accounting for 13.4% (PC1), 13.3% (PC2) and 10.3% (PC3) of the total variation: A) PC1 (X–axis) and PC2 (Y–axis), B) PC1 (X–axis) and PC3 (Y–axis) and C) PC2 (X–axis) and PC3 (Y–axis).

PCA results were in agreement with those of STRUCTURE (Fig 4). The first component (13.4% of the overall variation) clearly separated KHR, KKB, KSB and the rest of the sample localities (Fig 4A and 4B). The second component (13.3% of the overall variation) separated KHR, KKB and the others (Fig 4A and 4C); while the third component (10.3% of the overall variation) clearly differentiated WAK from the rest (Fig 4B and 4C). Hence, the analysis based on the three main components suggested five different groups (KHR, KKB, KSB, WAK and the rest of the samples). These groupings also were supported by results of the AMOVA analysis (*F*_CT_ = 0.005 and *p* < 0.001, *F*_SC_ = 0.001 and *p* = 0.273, Table 4). In contrast, grouping samples by geographical regions led to inconclusive results (*F*_CT_ = -0.000 and *p* = 0.620, *F*_SC_ = 0.003 and *p* < 0.001, Table 4).

**Table 4:** Analysis of Molecular Variance (AMOVA) in red sea bream for fifteen microsatellites.

Groups	Source of variation	Variance	Fixation index	% of variation	*p*-value
KHR vs. KSB vs. WAK vs. KKB vs. Rest					
	Among groups *F*_CT_	0.032	0.005	0.52	**<0.001**
	Among populations within groups *F*_SC_	0.003	0.001	0.06	0.273
	Within populations *F*_ST_	6.152	0.006	99.40	**<0.001**
Japan Sea vs. Pacific Ocean vs. Seto Inland Sea					
	Among groups *F*_CT_	-0.001	0.000	0	0.62
	Among populations within groups *F*_SC_	0.016	0.003	0.25	**<0.001**
	Within populations *F*_ST_	6.152	0.002	99.74	**<0.001**

Two independent analyses are presented, partitioning genetic variability among sampling location and among regions (seas). Bold *p*-values are statistically significant values.

The spatial analysis performed in BARRIER suggested three main barriers to gene flow around the genetically most divergent sampling locations (Fig 1). The two main barriers located around WAK (9 loci and 80% bootstrap support) and KKB (8 loci and 78% bootstrap support). A third barrier to gene flow was suggested around KTB; however, it presented weaker support (5 loci and 49% bootstrap). Current patterns of spatial genetic population structure observed in red sea bream in Japan were not significantly associated with geographic distances based on the results of the IBD analysis and partial Mantel test (slope = 0.039, r = -0.048, *p* = 0.337, data not shown). Excluding the pairwise value corresponding to the proximal sampling localities from Kanagawa Prefecture (KSB and KTB) turned the sign of the slope into a positive value; nevertheless, the regression remained statistically non-significant at the 5% level (data not shown).

Estimates of effective population sizes ranged between 48 in KHR to 614 in FKS (Table 5). The lowest estimates corresponded to KHR (*N*_e_ = 48), WAK (*N*_e_ = 126) and KKB (*N*_e_ = 163), which also showed the most divergent genetic profiles. In fact, only KHR and WAK gave finite confidence intervals.

**Table 5:** Estimates of effective population size (*N*_e_) and 95% confident intervals (95% CI) for red sea bream collections.

Sample	*N*_e_	95% CI
AIC	303	101-∞
FUK	410	124-∞
YAM	295	80-∞
FKS	614	124-∞
IWA	288	84-∞
KSB	491	120-∞
KTB	520	140-∞
KHR	48	34–77
WAK	126	74–337
EHI	283	92-∞
NAB	382	90-∞
FKK	312	90-∞
NGI	592	128-∞
OIT	357	113-∞
KKB	163	730-∞
KFA	505	111-∞

## Discussion

Our results based on fifteen microsatellite loci suggests weak, but consistent patterns of genetic divergence among red sea bream samples from coastal waters of Japan. The species appeared spatially structured in several patches with differentiated allelic composition, corresponding to areas receiving an important influx of fish of hatchery origin, either released intentionally [11, 14, 66] or from unintentional escapees [33, 67] and references therein. Due to the absence of information regarding the original genetic composition of the species before intensive red sea bream juvenile releases started, it is impossible to determine whether the current patchy pattern in population genetic structure occurred naturally or whether were it was induced by humans. Overall, our results suggest no direct correlation between the number of hatchery red sea bream juveniles released and any putative genetic effects associated with them. For example, despite the large number of juvenile releases accounted in Ehime Prefecture (Fig 2), EHI appeared genetically homogeneous to other sampling locations with different release histories (c.f., Table 3). Rather, the putative genetic effects of a particular stock enhancement program would be localized to specific areas depending upon many factors including the particular broodstock management practices of the program, the history of the releases, the oceanographic and geographical conditions of the area or the adaptive response of the hatchery-released offspring. In this regard, the differential genetic profiles among KKB, KSB and the rest of the samples (cf., Table 3, Figs 3 and 4) may reflect the results of the long-term and large-scale stock enhancement programs carried out in Kagoshima Bay and Sagami Bay [14, 66]. Another major finding of this study is that, in addition to local populations inhabiting semi-enclosed embayments, such as KKB and KSB [11, 25], large-scale releases (intentional or unintentional) also may have perturbed the genetic structure in open areas, e.g., WAK. Hence, current findings support the general concern about the potential harmful genetic and evolutionary effects of large-scale releases of cultured fish on natural populations [12, 13], and warn about the risk of conducting independent large-scale marine stock enhancement programs simultaneously on one species at different geographical locations. Future studies should clarify fitness performance of hatchery-released fish and demographic resiliency in heavily supplemented populations.

### Population genetic structure in relation to the major oceanic currents

Genetic comparisons between red sea bream samples separated by hundreds of kilometers indicated low but significant levels of genetic differentiation (overall *F*_ST_ = 0.002, *p* < 0.001). Disagreement with previous studies suggesting a large panmictic population in Japan [19, 26–28] can be related to the larger sample size, more microsatellite loci screened, and a larger geographic scope of sampling in the current study. The hatchery-released sample, KHR, presented the most distant allelic composition (Figs 3 and 4) and confirmed the loss of rare alleles commonly observed in stock enhancement programs [3, 12] and references therein, [18]. In addition to KHR, our analysis revealed particular gene pools at three sample localities (WAK, KKB and KSB) where no fish presented the characteristic deformity of the internostril epidermis of hatchery fish [39].

#### Seto Inland Sea and Pacific coast

Genotyping three microsatellite loci, Perez-Enriquez and Taniguchi [27] initially suggested the existence of two distinct stocks in Japan, one of them in the Japan Sea and the southwestern coast and a second one on the Pacific coast. However, in a more recent analysis using the same set of loci [28], they attributed the differences in allelic composition in specimens from Kochi and Wakayama Prefecture to disturbances originating from juvenile fish releases and the small size of the resident populations. Unfortunately, no sample from Kochi Prefecture was available for our analysis. Nevertheless, the particular genetic profiles of the fish from Wakayama, (WAK in Figs 3 and 4), and the fact that only KHR exhibited fewer alleles (Table 1) and a smaller genetically effective population size (Table 5) supports that explanation [26, 28]. The warm Kuroshio Current also may play a significant role in the particular genetic structure of red sea bream from Wakayama Prefecture. Although adult red sea bream have been shown to migrate relatively short distances [19] and references therein, [37], the strong northeastward Kuroshio Current may facilitate egg and larval drift both from the main southern as well as from smaller neighboring spawning grounds [19] and references therein, [33] towards the Seto Inland Sea of Japan. The proximity of the Kuroshio Current to the coast of Wakayama Prefecture [68] and the particular oceanography around the Kii Chanel, which connects the eastern part of the Seto Inland Sea to the open sea in the Pacific Ocean, may favor egg and larval retention [69]. However, intrusion of Kuroshio waters into the shelf and coastal waters of the Seto Inland Sea occur, and may originate “Kyucho”, or “sudden stormy currents” in Japanese [68, 70]. This process may cause significant damage and result in escape episodes from aquaculture facilities in the Seto Inland Sea [33, 67] and references therein. In addition, spontaneous spawning events of cultured fish from outdoor facilities in the area [71] also could have resulted in introgression of hatchery genotypes into wild populations.

Current results suggest a temporally stable pattern of genetic structure of red sea bream in Wakayama Prefecture [26, 28], where red sea bream appeared genetically divergent from the neighboring samples from the western (OIT) and the central part (EHI) of the Seto Inland Sea. These findings contrast with the pattern of population genetic structure reported for other fish species heavily stocked in the Seto Inland Sea of Japan [21, 72]. For both Japanese flounder [72] and sea chub (*Girella punctata*) [21], populations inhabiting the Seto Inland Sea diverged genetically from the coastal populations in the Pacific. In another recent study, Nakajima et al. [73] evaluated the temporal stability in patterns of genetic population structure in relation to the stock enhancement program conducted on Japanese Spanish mackerel (*Scomberomorus niphonius*) in the Seto Inland Sea, and reported no significant temporal changes in the diversity indices. However, most pairwise *F*_ST_ comparisons involving a post-release sample from the eastern part of the Seto Inland Sea were significant, including the comparison with the pre-release sample from the same area.

The strong Kuroshio Current also is likely to contribute to gene flow and genetic homogeneity among red sea bream populations along the Pacific coast (Figs 3 and 4). Previous studies conducted on different marine fishes and invertebrates have demonstrated high potential for egg and larval drift over hundreds of km along the Pacific coast of Japan [74, 75]. Interestingly, the Tokara Gap, situated around 28°N between the island of Kyushu and Taiwan, has been proposed to be a significant barrier to some organisms traveling with the Kuroshio Current, although once they cross it, they can spread rapidly onwards [68, 74]. In the northeastern part of Japan, the admixture potential of the cold Oyashio Current also was shown by Umino et al. [21], who suggested that *Girella punctata* larvae drifted almost 300 km from the spawning ground. The similarity in gene pools observed among red sea bream populations along the latitudinal range contrasts with results for Pacific herring (*Clupea pallasii*), another species subject to intensive long-term releases on the northern coasts of Japan [11, 76]. Heavily stocked wild southern populations of Pacific herring showed significant loss of rare alleles compared to un-stocked northern populations [76]. This reduction of genetic diversity was attributed to small population sizes and bottleneck effects due to the fact that the species was collected at the limit of its distribution range, and not to release activities [11]. In this study, the northern-most sample (IWA) was collected close to the limit of red sea bream distributional range (c.f. Fig 1, [29]); nevertheless, it exhibited similar levels of genetic variability to more southern samples (Table 1). Very large population sizes and high levels of gene flow may explain the high levels of genetic variability and low differentiation observed in red sea bream along the whole study area.

#### Southwest and Japan Sea

Similar to the Pacific coast, the genetic profiles of red sea bream in the Southwest and the Japan Sea coast appeared even less structured (*F*_ST_ = 0.001, *p* < 0.001; without KKB, *F*_ST_ = -0.000, *p* = 0.078), suggesting high levels of demic connectivity and more population admixture. The warm Tsushima Current can transport the early pelagic stages of several marine organisms; such as lobsters, argonauts and fishes [20] and references therein, [21], northeastwards over long distances. For example, for Japanese flounder, a species with a comparable pelagic phase duration to red sea bream [77], Kinoshita et al. [20] estimated the distance travelled with the currents to exceed 600 km. Thus, it seems likely that, similarly to the Kuroshio Current on the Pacific coast, the Tsushima Current may favor dispersal and connectivity between red sea bream populations along the western coast.

### Genetic disturbance in semi-enclosed areas with an intensive stocking history

Our analysis showed the distinct allelic composition of red sea bream inhabiting Kinko Bay in Kagoshima Prefecture (KKB) and Sagami Bay in Kanagawa Prefecture (KSB), two bays with an intense juvenile release history [11, 14, 66]. Concerns regarding the distinct genetic profiles between hatchery-released and native populations already have been emphasized [11, 19, 25]. While the open ocean and strong currents surrounding the Japanese archipelago favor dispersal and population connectivity [20, 21], the particular geographical and bathymetric features of Kinko Bay and Sagami Bay and the large number of hatchery fish released at these locations may have prompted disturbance of the original gene pool.

Kinko Bay presents a maximum depth of 237 m and a mouth width of 8.7 km. Oceanic drift was suggested to favor gene flow and admixture between red sea bream populations inhabiting the inner and outer part of the bay [23]. Earlier studies reported a significant loss of rare alleles [11, 23] and mitochondrial haplotype richness [24] in the inner bay population. The erosion of genetic diversity was ascribed to density-dependent effects associated with large-scale releases and to the small broodstock used to produce the juveniles over several generations. All analyses that we conveyed here (Table 3, Figs 3 and 4) highlighted the particular genetic composition of red sea bream from Kinko Bay (KKB). Moreover, estimates of effective population size suggested KKB as one of the smallest effective population sizes (Table 5); although the large CI requires cautious interpretation of the estimates [62]. Reduction in genetic diversity, survival, growth and reproductive fitness are some major effects attributed to hatchery releases [12, 78, 79]. At present, no data on fecundity, growth rate, age-at-maturity or reproductive success is available for red sea bream in KKB. As already mentioned, the absence of genetic information prior to commencement of juvenile releases also impedes drawing definite conclusions regarding the putative long-term genetic erosion of the local population [3, 12, 13]. Yet, current results may call into question results of a previous review suggesting no decline in population fitness [11, 24].

Sagami Bay (KKB), and to a lesser extent the neighboring Tokyo Bay (KTB), appeared as another area where red sea bream showed a distinct genetic composition. In contrast to Kinko Bay, Sagami Bay reaches almost 1500 m in depth with a narrow continental shelf and sudden drop-off, and presents a wide mouth to the open sea which offers possibilities for gene flow. Meanwhile, the eastern side of Sagami Bay is connected to Tokyo Bay and likely receives an influx of fish from this semi-enclosed bay. Population dynamics of red sea bream in these interconnected bays is highly influenced by juvenile releases, which may comprise up to 74% of commercial landings [66]. In a recent study, we observed significantly lower genetic diversity in hatchery-produced juveniles, both pre- and post-release, compared to resident populations in Sagami Bay and Tokyo Bay, and anticipated putative deleterious effects in case of wild and hatchery fish interbreeding (for details, see in [25]).

Disparity in the genetic profiles of populations inhabiting semi-enclosed areas and with an intensive stocking history is not exclusive to red sea bream. Studies conducted with heavily released Japanese turban shell (*Turbo* (*Batillus*) *cornutus*) [80] and black sea bream (*Acanthopagrus schlegelii*) [81] already have shown distinct genetic profiles in some local embayments subject to stocking activities. However, our knowledge regarding the long-term effects of marine stock enhancement and augmentation programs is still very limited [11, 12]. In fact, while enclosed areas are extensively targeted in order to achieve high return rates and justify the cost-efficiency of the releases, only a small portion of marine stock enhancement programs carried out in recent years included a genetic evaluation [12] and references therein. Moreover, in a review of the long-term genetic effects of stock enhancement programs, Kitada et al. [11] concluded that they are small, localized and temporary. However, the current study suggests that intensive hatchery releases (either as part of stock enhancement programs or from unintentional aquaculture escapees) may perturb the gene pool of wild stocks in open areas [26, 28]. Previous studies on salmonids also have shown that the loss of genetic variability and homogenizing effects associated to stocking [8, 10] and fish escapees [79] can have a lasting impact on population genetic structuring and compromise the evolutionary potential of the species. Future studies should deepen our understanding of adaptive fitness in red sea bream. Investigations on other species and programs also should clarify the magnitude and putative long-term effects of hatchery releases on marine species.
